# Spectrum Correction Using Modeled Panchromatic Image for Pansharpening

**DOI:** 10.3390/jimaging6040020

**Published:** 2020-04-06

**Authors:** Naoko Tsukamoto, Yoshihiro Sugaya, Shinichiro Omachi

**Affiliations:** Graduate School of Engineering, Tohoku University, Sendai, Miyagi 980-8579, Japan; sugaya@iic.ecei.tohoku.ac.jp (Y.S.); machi@ecei.tohoku.ac.jp (S.O.)

**Keywords:** pansharpening, spectrum correction, intensity correction, model, relative spectral response graph, IKONOS

## Abstract

Pansharpening is a method applied for the generation of high-spatial-resolution multi-spectral (MS) images using panchromatic (PAN) and multi-spectral images. A common challenge in pansharpening is to reduce the spectral distortion caused by increasing the resolution. In this paper, we propose a method for reducing the spectral distortion based on the intensity–hue–saturation (IHS) method targeting satellite images. The IHS method improves the resolution of an RGB image by replacing the intensity of the low-resolution RGB image with that of the high-resolution PAN image. The spectral characteristics of the PAN and MS images are different, and this difference may cause spectral distortion in the pansharpened image. Although many solutions for reducing spectral distortion using a modeled spectrum have been proposed, the quality of the outcomes obtained by these approaches depends on the image dataset. In the proposed technique, we model a low-spatial-resolution PAN image according to a relative spectral response graph, and then the corrected intensity is calculated using the model and the observed dataset. Experiments were conducted on three IKONOS datasets, and the results were evaluated using some major quality metrics. This quantitative evaluation demonstrated the stability of the pansharpened images and the effectiveness of the proposed method.

## 1. Introduction

The optical sensor of an Earth observation satellite receives radiances in the visible to infrared regions of the electromagnetic spectrum. The sensor simultaneously receives two kinds of data; the multi-spectral (MS) image with high spectral resolution and low spatial resolution, and a panchromatic (PAN) image with high spatial resolution and low spectral resolution. Satellites with such optical sensors include the IKONOS, QuickBird, GeoEye, and WorldView. Satellite data are widely used for various purposes such as change detection, object detection, target recognition, background for map application, and visual image analysis. Pansharpening is an image processing technique that generates high-spatial and high-spectral-resolution MS images using the spatial information from the PAN image and the spectral information from the MS images. It can be used for preprocessing in the data analysis and applications of satellite-image processing described above. The pansharpening methods can be divided into four categories: component substitution (CS), multi-resolution analysis (MRA), machine learning, and the hybrid methods. The CS methods substitute the spatial information of the MS images by the spatial information from the PAN image and then generate the MS images with high spatial resolution. This category of methods includes several techniques such as the intensity–hue–saturation (IHS) transform [1], principal component analysis [2], Gram–Schmidt (GS) transform [3,4], and the Brovey transform [2]. For these techniques, it is known that the difference in the spectral characteristics between the replaced spatial component and the original spatial component gives rise to spectral distortion. The MRA class extracts the spatial information from the PAN image and then adds it to the MS image. For these methods, several techniques such as the decimated wavelet transform [5], undecimated wavelet transform [6], a “trous” wavelet transform [7], Laplacian pyramid [8], curvelet transform [9], contourlet transform [10], nonsubsampled contourlet transform [11], and shearlet transform [12,13] are used to extract the detailed spatial information. Although these methods increase the quality of the spectral information, ringing artifacts called spatial distortion may occur. Machine learning methods use techniques such as dictionary learning, sparse modeling [14], deep learning [15], and the Bayesian paradigm [16]. The performance of these methods depends on the amount of the available data, and their computational complexity is greater than that of the other methods. For example, sparse modeling incurs a significant computational cost for creating a dictionary. The hybrid methods combine several techniques to exploit their advantageous features and therefore can achieve higher performance than the methods in other classes. Recently, machine learning techniques have been widely used. Wang et al. [17] proposed a method using sparse representation and a convolution neural network. Fei et al. [18] and Yin [19] proposed an improved Sparse Representation-based details injection (SR-D) [14]. For the CS-based category, Imani [20] proposed a method that removes the noise and redundant spatial features to improve the band-dependent spatial detail (BDSD) [21].

Many methods have been proposed over the past several decades in order to reduce the spectral distortion and to enhance the spatial resolution [22,23,24] and in particular, pansharpening includes a process for correcting the image intensity. Two types of techniques are used for this process: the first uses the relative spectral response graph, and the second uses an intensity model based on the observed images. However, the numerical image quality of these methods depends on the image dataset, and it is difficult to obtain consistent results.

In this study, we propose a technique for correcting the intensity based on the IHS method. The IHS method is a known pansharpening method and substitutes the intensity of the red–green–blue (RGB) images with the intensity of the PAN images. Since the intensity of the pansharpened (PS) image is replaced by that of the PAN image, it contains a high level of spatial information. However, the PS image exhibits spectral distortion (i.e., color distortion) because of the differences in the spectral characteristics between the intensities of the RGB and PAN images [25]. To address this drawback, several methods have been proposed. Tu et al. [26] proposed a generalized IHS (GIHS) transform that transforms the IHS into a simple linear transform. Since GIHS can accelerate the calculations, it has been frequently used as a framework for the development of other methods focusing on correcting the intensity. Tu et al. [26] presented a fast IHS that corrects the intensity using the mean value of the intensity of the MS images and also presented a simple spectral-adjustment IHS method (SAIHS) [27] that corrects the intensity of the green and blue bands by exploring the best value from 92 IKONOS images. Tradeoff IHS is a method that controls the tradeoff of the spectral characteristics between the intensities of the RGB and PAN images [28]. Choi et al. [29] presented an improved SAIHS (ISAIHS) that calculates the intensity correction coefficients of the red and near-infrared (NIR) bands using 29 IKONOS images in addition to SAIHS. These approaches have the advantage that strong outliers are not generated because the correction coefficient is obtained from the images as a constant. However, the values of the constant may not be optimal for the processed image. Audicana et al. [30] proposed an expanded fast IHS with the spectral response function (eFIHS-SRF) method to correct the intensity of the PS image using the mean value of the MS images and the fraction of the photons detected by the sensors (i.e., the MS and PAN sensors). In this method, the correction is performed using the correction coefficient obtained from the relative spectral response graph and the observation data. However, the obtained results differ depending on the processed data. Garzelli et al. [21] presented the BDSD method that applies the correction coefficients calculated by the minimum-variance-unbiased estimator [31] to the MS images. The practical replacement adaptive CS (PRACS) [32] calculates the correction coefficients using high-frequency information and the characteristics of the MS images for each image dataset. The fusion approach (IHS-BT-SFIM) proposed by Tu et al. [33] calculates the intensity correction coefficients using the modeled low-spatial-resolution intensity of the MS images.

These methods use either unique or non-unique correction coefficients. In pansharpening, spectral distortion may be caused by three different effects: the relative spectral response of the sensors [27], aging of the optical and electronic payload [34], and the observation conditions [35]. Since the observation conditions differ for each dataset, the correction coefficients must be calculated separately for each image dataset. Even in the conventional methods, the correction coefficient is obtained from the intensity modeled based on the image. However, the use of a model formula obtained by combining the intensity with and without color information has not been considered. We estimate the PAN image without color information using the intensity of the RGB image and the intensity of the NIR image, and perform a detailed correction including the relative spectral response and observation conditions at each intensity of the RGB image with color information. In this study, we propose a novel model for low-spatial-resolution PAN images using MS images. Compared to other related methods for intensity correction, our method showed consistently good performance in terms of the numerical image quality. Therefore, it was concluded that unlike for the methods based on IHS, the proposed method can reduce spectral distortion and obtain results that are independent of the processed image.

Note that there are multiple satellites that have sensors whose characteristics are similar to those of IKONOS, such as Quick Bird and GeoEye. Therefore, experiments have been conducted on IKONOS images in many studies in the literature. Our proposed method can also be applied to those satellite images.

## 2. Materials and Methods

### 2.1. Image Datasets

The three image datasets from the IKONOS used for the experiments are listed in Table 1. The first was collected in May 2008 and covers the city of Nihonmatsu, Japan. The second was collected in May 2006 and covers the city of Yokohama, Japan. Both the Nihonmatsu and Yokohama datasets were provided by the Japan Space Imaging Corporation, Japan. The third dataset covering Mount Wellington in Hobart, Tasmania in Australia was collected in February 2003 and was provided by Space Imaging, LLC. These datasets have PAN and MS images with the spatial resolutions of 1 m and 4 m, respectively. The original dataset contains: a PAN image with 1024 × 1024 pixels and MS images with 256 × 256 pixels for the Nihonmatsu region, a PAN image with 1792 × 1792 pixels and MS images with 448 × 448 pixels for the Yokohama region, and a PAN image with 12,112 × 13,136 pixels and MS images with 3028 × 3284 pixels for the Hobart region in Tasmania. 

To evaluate the quality of the PS image, we experimented with the test images and original images according to the Wald protocol [36]. The test images were used to evaluate the numerical image quality, and the original images were used as reference images for numerical and visual evaluation. We regard the original images as ground truth images. The spatial resolution of the test PAN image was reduced from 1 to 4 m and that of the test MS image was reduced from 4 to 16 m. Hence, the test image datasets have a PAN image with 256 × 256 pixels and MS images with 64 × 64 pixels for the Nihonmatsu region, a PAN image with 448 × 448 pixels and MS images with 112 × 112 pixels for the Yokohama region, and a PAN image with 3028 × 3284 pixels and MS images with 757 × 821 pixels for the Hobart region in Tasmania.

### 2.2. Proposed Method

It is considered that a high-resolution PAN image can be estimated from the corresponding high-resolution MS images. However, it is not obvious how to combine the MS images, and it may differ depending on the spectral response and observation conditions. Based on this idea, we propose a novel technique for correcting the intensity that can reduce the spectral distortion in each image by modeling the PAN image. The technique does not require detailed knowledge of sensor characteristics; in other words, it only requires the image dataset. The procedure of the proposed method includes a technique for estimating the intensity correction coefficients and also a technique for image fusion. The former method first models the PAN image with a low spatial resolution, using the MS images, and the coefficients are then calculated using the method of least squares in the comparison between the PAN image and the modeled PAN image. The latter method calculates the high-spatial-resolution intensity of the RGB image and then generates a PS image using GIHS.

#### 2.2.1. Notation

$I^{high}\left(i\right)$ and $I^{low}\left(i\right)$ denote the *i*-th pixel intensities of the high- and low-spatial resolution RGB images, respectively. $PAN^{high}\left(i\right)$ and $PAN^{low}\left(i\right)$ denote the *i*-th pixel high- and low-spatial resolution, respectively. $MS_{b\in B}^{low}\left(i\right)$ denotes the *i*-th pixel low-spatial resolution MS image in the *b* band and B={red,grn,blu,nir}; *red*, *grn*, *blu*, and *nir* are the red, green, blue, and NIR, respectively. |B| denotes the total number of MS bands. ‘·’ denotes the scalar multiplication of matrices, ‘×’ denotes multiplication, and ‘∗’ denotes a matrix product. *N* denotes the total number of the pixels in an image.

#### 2.2.2. High-Spatial-Resolution Intensity of the RGB Image

The intensity of the RGB image is calculated by the IHS transform and is represented by Equation (1).

(1) $$I^{low}\left(i\right)=\frac{MS_{red}^{low}\left(i\right)+MS_{grn}^{low}\left(i\right)+MS_{blu}^{low}\left(i\right)}{3}$$

The intensity ratio between the PAN and RGB images is the same for the high- and low-spatial resolutions. Thus, if we have both PAN and RGB images with high- and low-spatial resolutions with the same number of pixels, this intensity relationship can be expressed as:

(2) $$I^{high}\left(i\right):PAN^{high}\left(i\right)=I^{low}\left(i\right):PAN^{low}\left(i\right)$$

The dimensions of the PAN and MS images used for the above process are the same as those of the MS images before processing, ${\mathbb{R}}^{P\times P}$. The PAN image is generated by down-sampling with bicubic interpolation.

To obtain an expression for the high-spatial-resolution intensity of the RGB image, Equation (2) can be rewritten as

(3) $$I^{high}\left(i\right)=\frac{PAN^{high}\left(i\right)}{PAN^{low}\left(i\right)}\times I^{low}\left(i\right)$$

#### 2.2.3. Low-Spatial-Resolution PAN Image Model

**Figure 1:**
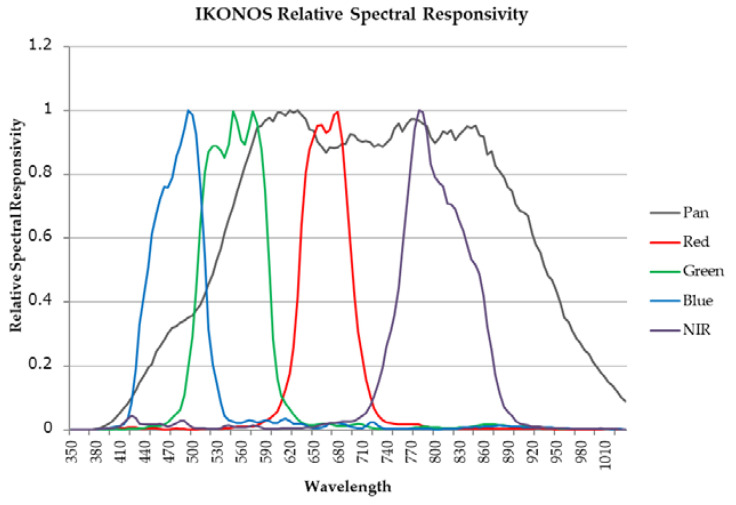
IKONOS relative spectral response graph.

Since the observation wavelength range of the PAN sensor includes the wavelength ranges of all of the MS sensors, we modeled the low-spatial-resolution PAN image using low-spatial-resolution MS images. Based on the relative spectral response graph of IKONOS [37] depicted in Figure 1, we noticed the following features:

1. The relative spectral response of the PAN band shows low sensitivity from the blue to green bands [27].
2. The relative spectral responses of the PAN band show low sensitivity in some regions of the red and NIR bands.
3. The relative spectral responses of the red, green, blue, and NIR bands partly overlap with those of their neighboring bands [4,27].
4. The PAN band includes the NIR band.

We design the low-spatial-resolution PAN image model including the above features as follows:

(4) $$PAN^{low}\left(i\right)=I^{low}\left(i\right)+\alpha \cdot MS_{nir}^{low}\left(i\right)-\beta \cdot MS_{blu}^{low}\left(i\right)-\gamma \cdot MS_{grn}^{low}\left(i\right)-\xi \cdot MS_{red}^{low}\left(i\right)$$

where α, β, γ, and ξ denote the correction coefficients of the NIR, blue, green, and red bands, respectively. For the right-hand side of Equation (4), the third and fourth terms are affected by feature 1, the second and fifth terms are affected by feature 2, the second to fifth terms are affected by feature 3, and the second term is affected by feature 4. The first and second terms on the right-hand side of Equation (3) estimate the PAN image using the intensities of the RGB and NIR images. The third, fourth, and fifth terms are considered to be the overflowing and overlapping parts of the MS bands in the relative spectral response graph. The overflowing part of the NIR band is included in the coefficient α.

#### 2.2.4. Estimation of the Intensity Correction Coefficient

The high- and low-spatial images observed under the same conditions have similar intensity characteristics, and the relationship between the high- and low-spatial resolution PAN images can be expressed as $PAN^{high}\left(i\right)≃PAN^{low}\left(i\right)$. Therefore, the intensity correction coefficients are obtained when the sum of the differences between the high- and low-spatial resolution PAN images reaches the minimum value, as expressed by the root mean square error that is computed as:

(5) $$\underset{\alpha ,\beta ,\gamma ,\xi }{\mathrm{argmin}}\sqrt{\frac{1}{N}\sum_{i=1}^{N}{\left(PAN^{high}\left(i\right)-PAN^{low}\left(i\right)\right)}^{2}}\;s.t.\;\alpha ,\beta ,\gamma ,\xi \geq 0$$

Equation (5) is used with the observed data—that is, the PAN and MS images, because the observed data include the relative spectral response of the sensors and the observation conditions. In practice, we use Equation (6) instead of Equation (5). The term $\left(PAN^{high}\left(i\right)-PAN^{low}\left(i\right)\right)$ in Equation (5) can be rearranged as $\left(PAN^{high}\left(i\right)-I^{low}\left(i\right)\right)+\left(-\alpha \cdot MS_{nir}^{low}\left(i\right)+\beta \cdot MS_{blu}^{low}\left(i\right)+\gamma \cdot MS_{grn}^{low}\left(i\right)+\xi \cdot MS_{red}^{low}\left(i\right)\right)$.

To express the above formula in matrix representation, we define

$$A=\left[\begin{array}{cccc} -MS_{nir}^{low}\left(1\right) & MS_{blu}^{low}\left(1\right) & MS_{grn}^{low}\left(1\right) & MS_{red}^{low}\left(1\right)\\ \vdots & \vdots & \vdots & \vdots \\ -MS_{nir}^{low}\left(N\right) & MS_{blu}^{low}\left(N\right) & MS_{grn}^{low}\left(N\right) & MS_{red}^{low}\left(N\right) \end{array}\right]$$

$$c=\left[\begin{array}{c} \alpha \\ \beta \\ \gamma \\ \xi \end{array}\right],d=\left[\begin{array}{c} I^{low}\left(1\right)-PAN^{high}\left(1\right)\\ \vdots \\ \vdots \\ I^{low}\left(N\right)-PAN^{high}\left(N\right) \end{array}\right],\;A\in {\mathbb{R}}^{N\times \left|B\right|},\;c\in {\mathbb{R}}^{\left|B\right|},\;d\in {\mathbb{R}}^{N}\;$$

We compute the intensity correction coefficients using the least square method, as described in Equation (6):

(6) $$\underset{c}{\mathrm{argmin}}{\left\|A*c-d\right\|}_{2}^{2}\;\;s.t.\;\;c\geq 0.$$

#### 2.2.5. Fusion Process

**Figure 2:**
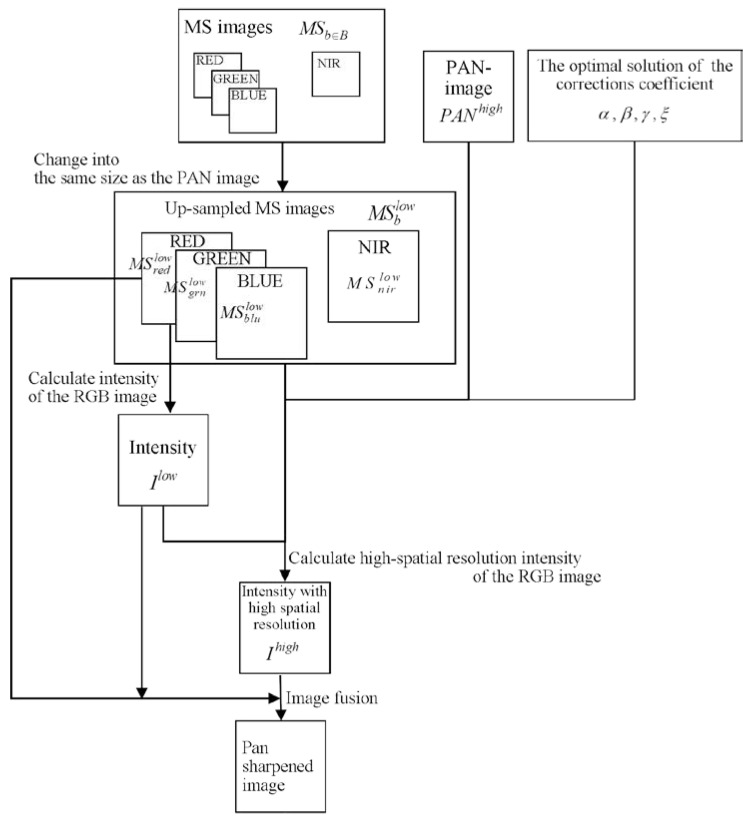
Fusion process diagram of the proposed method.

The fusion process generates a PS image with GIHS, using the intensity correction coefficients and the observed images. Since GIHS is a simple fusion technique, it is the optimal technique for the evaluation of the image quality performance. The validity of the GIHS formula was rigorously proved for RGB images in Tu et al. [26], and the extension to NIR images has been derived from the form of the equation. Therefore, this time we applied it to RGB images. Figure 2 shows the procedures of the fusion process, which are also listed below:

1. Change the MS images into the same size as that of the PAN image using bicubic interpolation, and produce enlarged MS images $MS_{b\in B}^{low}$.
2. Calculate the enlarged low-spatial-resolution intensity $I^{low}$ from $MS_{b\in \left\{red,grn,blu\right\}}^{low}$, as expressed by Equation (1).
3. Calculate the high-spatial-resolution intensity $I^{high}$ of $MS_{b\in \left\{red,grn,blu\right\}}^{low}$ using the estimated correction coefficients, $MS_{b\in \left\{red,grn,blu\right\}}^{low}$, $I^{low},$ and $PAN^{high}$ using Equations (3) and (4).
4. Synthesize the PS image from $I^{low}$, $MS_{b\in B}^{low},$ and $I^{high}$ with GIHS, as expressed by Equation (7).
  (7) $$\left[\begin{array}{c} MS_{red}^{PS}\left(i\right)\\ MS_{grn}^{PS}\left(i\right)\\ MS_{blu}^{PS}\left(i\right) \end{array}\right]=\left[\begin{array}{c} MS_{red}^{low}\;\left(i\right)+I^{high}\left(i\right)-I^{low}\left(i\right)\\ MS_{grn}^{low}\;\left(i\right)+I^{high}\left(i\right)-I^{low}\left(i\right)\\ MS_{blu}^{low}\;\left(i\right)+I^{high}\left(i\right)-I^{low}\left(i\right) \end{array}\right]$$

## 3. Results

### 3.1. Experimental Setup

The procedure of the proposed method used the image sizes listed in Table 2. Let the sizes of the original PAN and MS images be $RP_{row}\times RP_{col}$ and $P_{row}\times P_{col}$, respectively, where R is the ratio of the number of vertical and horizontal pixels of the PAN image relative to that for the MS image. Then, the size of the down-sampled test PAN image and test MS image used for the estimation intensity correction coefficients in Table 2 is $\frac{P_{row}}{R}\times \frac{P_{col}}{R}$, and the size of the Test PAN image and the up-sampled test MS image used for image fusion is $P_{row}\times P_{col}$. The test image was generated by bicubic spline interpolation. Due to the possible loss of data, the procedure for the estimation of the intensity correction coefficients does not use up-sampling. The correction coefficients were determined by solving Equation (5) using the non-negative least-squares method. The correction coefficient is the value for which the closest agreement is obtained between the spectral characteristics of the high-resolution PAN image and those of the low-resolution PAN image. The bicubic interpolation was used for down-sampling and up-sampling of the images used for the experiment of the proposed method shown in Table 2. For a fair comparison, the up-sampling of the MS images of the related works was also carried out using bicubic interpolation.

### 3.2. Image Quality Metric

#### 3.2.1. Notation

$O_{b}\left(i\right)$ and $\bar{O_{b}}$ denote the *i*-th pixel value of the *b*-band reference image and its mean value, respectively, and $PS_{b}\left(i\right)$ and $\bar{PS_{b}}$ denote the *i*-th pixel value of the *b*-band PS image and its mean value, respectively. N and |B| are the total number of pixels in the entire image for each band and the number of bands in the PS image, respectively. $\sigma_{O_{b}}$ and $\sigma_{PS_{b}}$ are the variances of the reference and PS images in the *b*-band, respectively, and $\sigma_{O_{b,PS_{b}}}$ denotes the covariance of the reference and PS images in the *b*-band. *h* and *l* denote the spatial resolution of the PAN and MS images, respectively.

#### 3.2.2. Numerical Quality Metrics

To evaluate the numerical image quality, we employed four metrics: the correlation coefficient (CC), university image quality index (UIQI) [38], *erreur relative globale adimensionnelle de synthese* (ERGAS) [39], and the spectral angle mapper (SAM) [40]. All of the metrics measure the spectral distortion, and UIQI, ERGAS, and SAM are global metrics.

The CC measures the correlation between the images and ranges from –1.0 to 1.0. A CC value closer to 1.0 implies a stronger correlation between the spectral information of the PS image and the original image. The CC is given by

(8) $$\begin{array}{c} \mathrm{CC}=\frac{1}{\left|B\right|}\times \underset{b\in B}{\sum }CC_{b}\\ {\mathrm{CC}}_{b}=\frac{\sum_{i=1}^{N}\left(O_{b}\left(i\right)-\bar{O_{b}}\right)\times \left(PS_{b}\left(i\right)-\bar{PS_{b}}\right)}{\sqrt{\sum_{i=1}^{N}{\left(O_{b}\left(i\right)-\bar{O_{b}}\right)}^{2}}\times \sqrt{\sum_{i=1}^{N}{\left(PS_{b}\left(i\right)-\bar{PS_{b}}\right)}^{2}}} \end{array}$$

UIQI [38] comprehensively measures the value of the loss of correlation, intensity distortion, and contrast distortion. The loss of correlation measures the degree of the linear correlation between the images. The intensity distortion measures the closeness of the mean intensity values of the images. Contrast distortion measures the similarity of the contrasts of the images. These values range from –1.0 to 1.0. A UIQI value closer to 1.0 implies smaller values of the loss of correlation, intensity distortion, and contrast distortion, so that a higher UIQI value corresponds to a higher quality of the PS image. UIQI is given by

(9) $$\begin{array}{c} \mathrm{UIQI}=\frac{1}{\left|B\right|}\times \underset{b\in B}{\sum }UIQI_{b}\\ {\mathrm{UIQI}}_{b}=\frac{\sigma_{O_{b,PS_{b}}}}{\sigma_{O_{b}}\cdot \sigma_{PS_{b}}}\times \frac{2\cdot \bar{O_{b}}\cdot \bar{PS_{b}}}{{\left(\bar{O_{b}}\right)}^{2}+{\left(\bar{PS_{b}}\right)}^{2}}\times \frac{2\cdot \sigma_{O_{b}}\cdot \sigma_{PS_{b}}}{\sigma_{O_{b}}{}^{2}+\sigma_{PS_{b}}{}^{2}} \end{array}$$

ERGAS [39] measures the global image quality with a lower ERGAS value corresponding to a higher spectral quality of the PS image, and it is given by

(10) $$\begin{array}{c} \mathrm{ERGAS}=100\times \frac{h}{l}\times \sqrt{\frac{1}{\left|B\right|}\times \underset{b\in B}{\sum }\left(\frac{{\left(RMSE_{b}\right)}^{2}}{{\left(\bar{PS_{b}}\right)}^{2}}\right)}\\ {\mathrm{RMSE}}_{b}=\sqrt{\frac{1}{N}\times \overset{N}{\underset{i=1}{\sum }}{\left(O_{b}\left(i\right)-PS_{b}\left(i\right)\right)}^{2}} \end{array}$$

${\mathrm{RMSE}}_{b}$ is the root-mean-square error between the reference image and the PS image in the *b*-band.

SAM [40] measures the global spectral distortion with the value closer to 0.0 corresponding to weaker spectral distortion, and is given by

(11) $$\begin{array}{c} \mathrm{SAM}=\frac{1}{N}\overset{N}{\underset{i=1}{\sum }}SAM\left(i\right)\\ \mathrm{SAM}\left(i\right)=\cos^{-1}\left(\frac{\sum_{b\in B}O_{b}\left(i\right)\times PS_{b}\left(i\right)}{\sqrt{\sum_{b\in B}{\left(O_{b}\left(i\right)\right)}^{2}}\times \sqrt{\sum_{b\in B}{\left(PS_{b}\left(i\right)\right)}^{2}}}\right) \end{array}$$

### 3.3. Experimental Results

The intensity correction coefficients were estimated using Equation (5). Table 3 lists the estimated intensity correction coefficients for the three datasets, where α represents the fraction of the NIR included in the PAN image, and β,  γ,  ξ are the fractions of the image where the intensity of the RGB image that does not match the PAN image. 

**Figure 3:**
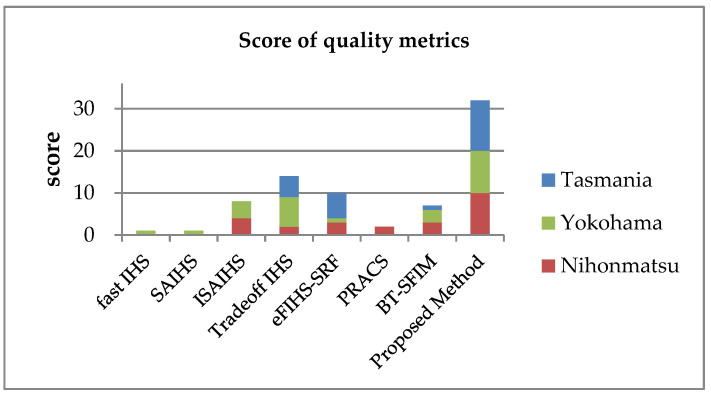
Quality metric result rank in three tested datasets: the score of the quality metrics is expressed as a cumulative bar chart.

The PS image of the proposed method was compared to those obtained by the related methods for intensity correction, namely fast IHS [26], SAIHS [27], ISAIHS [29], Tradeoff IHS [28], eFIHS-SRF [30], PRACS [32], and Brovey Transform-Smoothing-Filter-based Intensity Modulation (BT-SFIM) [33]. PRACS [32] was performed using the code developed by Vivone et al. [23]. The detailed parameters of the existing methods were as follows: the weight parameter for SAIHS was (Green,Blue) = (0.75,0.25), the weight parameters for ISAIHS were (Red,Green,Blue,NIR) = (0.3,0.75,0.25,1.7), the tradeoff parameter for the Tradeoff IHS was 4.0, the fraction of the number of photons in the MS band detected by the PAN sensor of eFIHS-SRF was 0.8, and the mean filter kernel size and the weight parameter for BT-SFIM were 7×7 and (Red,Green,Blue,NIR) = (0.26,0.26,0.122,0.375), respectively. The numerical image quality was evaluated using the CC, UIQI [38], ERGAS [39], and SAM [40] metrics. The sliding window size of UIQI was 8×8. Table 4 and Table 5 summarize the image quality results for CC and UIQI, and for ERGAS and SAM, respectively. It is observed that with the exception of the image of Tasmania, ISAIHS gave good results. The proposed method gave the best UIQI and ERGAS values for all of the images. The SAM values of eFIHS-SRF and the proposed method are not correlated with the size of the processed image, while the values obtained by the other methods decrease with the increasing number of pixels in the processed image. The Tradeoff IHS and the proposed method gave consistently good results for all of the images. Figure 3 shows the ranking of the quality metric results for the seven methods. Here, for each test image, the best result is worth three points, the second-best result is worth two points, and the third-best result is worth one point. The maximum possible number of points is 36, which is obtained when a method has the best values for all of the metrics. BT-SFIM uses the coefficients estimated from a relative spectral response graph and does not give good results. eFIHS-SRF using the coefficients estimated by the relative spectral response graph is the second-best method after Tradeoff IHS. In contrast, ISAIHS and Tradeoff IHS use the coefficients estimated from large image datasets and gave good results. These results show that techniques using image datasets tend to perform better than those using the relative spectral response graph. This demonstrates the need to consider other observation conditions in addition to the spectral response graphs, and it can be concluded that the observation dataset contains this information.

**Figure 4:**
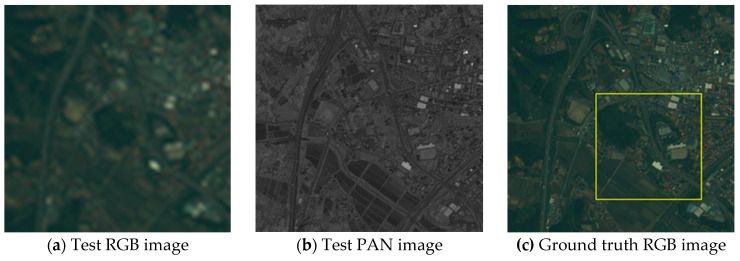

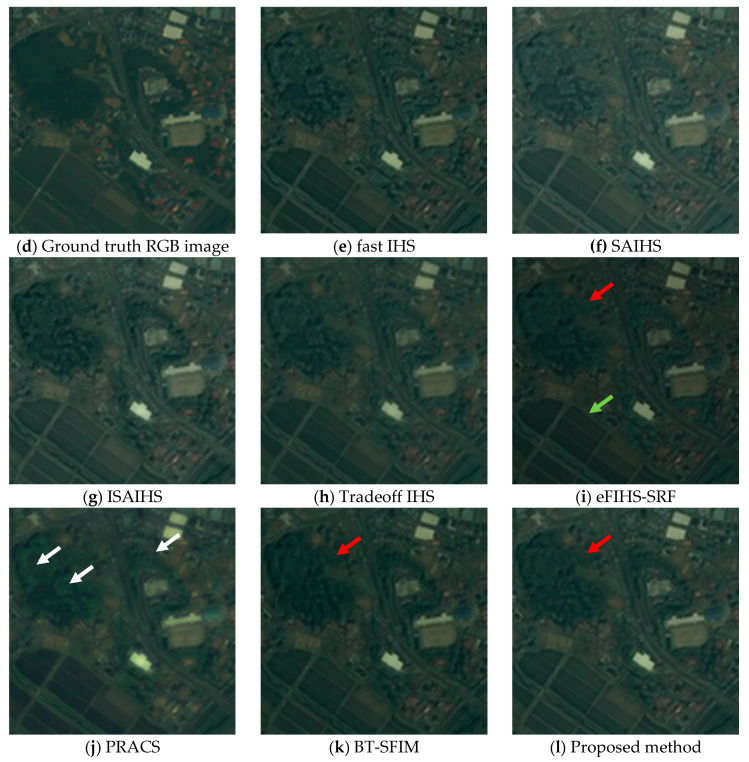
Visual comparison of test Nihonmatsu images: (**a**) Test red–green–blue (RGB) image; (**b**) Test PAN image; (**c**) Ground truth RGB image (i.e., Original RGB image); (**d**) Ground truth RGB image of the yellow box area in (c); (**e–l**) PS images generated by various methods corresponding to the area of (d); The red arrow indicates where the forest colors are well reproduced. The green arrow indicates where the color of the rice field is dark. White arrows indicate where the green tones are light.

**Figure 5:**
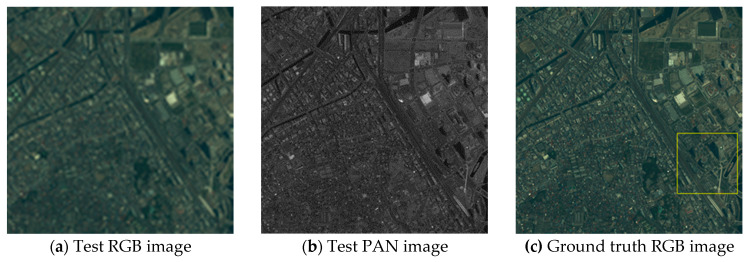

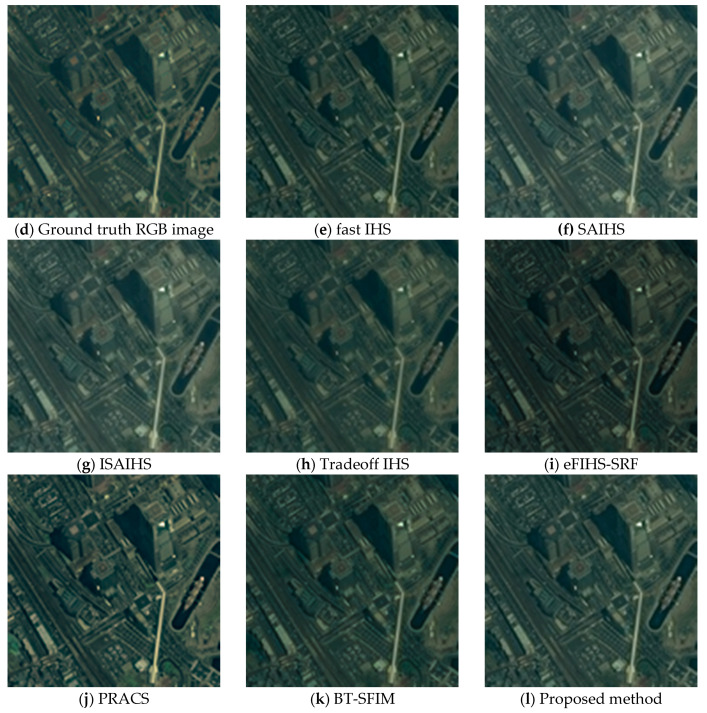
Visual comparison of test Yokohama images: (**a**) Test RGB image; (**b**) Test PAN image; (**c**) Ground truth RGB image (i.e., Original RGB image); (**d**) Ground truth RGB image of the yellow box area in (c); (**e–l**) PS images generated by various methods corresponding to the area of (d).

**Figure 6:**
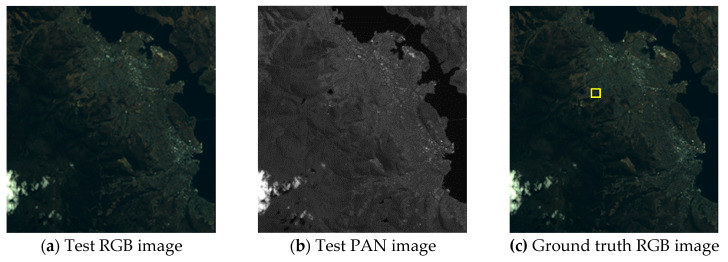

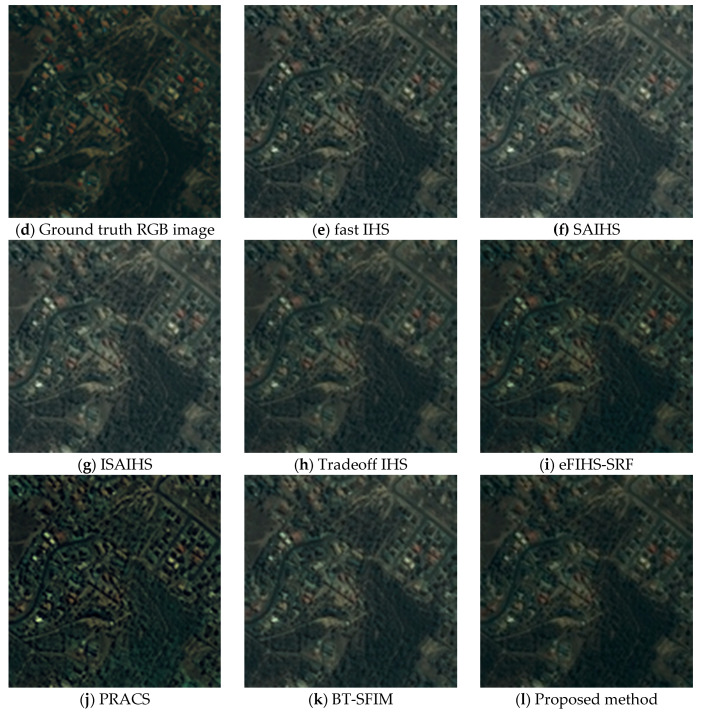
Visual comparison of test Tasmania images: (**a**) Test RGB image; (**b**) Test PAN image; (**c**) Ground truth RGB image (i.e., Original RGB image); (**d**) Ground truth RGB image of the yellow box area in (c); (**e–l**) PS images generated by various methods corresponding to the area of (d).

Visual analyses are shown in Figure 4 Figure 5 and Figure 6. In this evaluation, the PS images generated from the test images were compared to the ground truth RGB images. In Figure 4 and Figure 5, (d)–(l) are the expanded images corresponding to the area surrounded by the yellow box in (c). As shown in Figure 4, eFIHS-SRF (Figure 4i), BT-IHS (Figure 4k), and the proposed method (Figure 4l) reproduced the color tone of the forest (indicated by red arrows), while the color of the rice field in Figure 4i for eFIHS-SRF was darker (indicated by green arrows). The green component of PRACS (Figure 4j) was brighter than expected (indicated by white arrows). As shown in Figure 5, SAIHS (Figure 5f) and ISAIHS (Figure 5g) images were generally brighter, and the eFIHS-SRF image (Figure 5i) was darker. In Figure 6, eFIHS-SRF (Figure 6i) and the proposed method (Figure 6l) reproduced the overall color tone, while the PRACS (Figure 6j) image had a brighter appearance than the images obtained using the other methods, which were generally whitish. In summary, for the proposed method (Figure 6l), the color tone of the whole image was consistent with the ground truth image for all images, and the resolution was also good. The green component of the PRACS image (Figure 6j) is brighter. The results obtained by the other methods differed depending on the image.

## 4. Discussion

The experimental results show that for SAM, only the proposed method and eFIHS-SRF for which the coefficients are estimated by the relative spectral response graph do not depend on the size of the processed image. This indicates that the overall color was not destroyed. In other words, there is little spectral distortion. Tradeoff IHS performs well on average for all images; however, it does not exhibit best fitting for the processed images. Since each method has advantages and disadvantages, we calculated the ranking of the quality metric. According to the results of this ranking, the proposed method is the best, followed by Tradeoff IHS and eFIHS-SRF. Tradeoff IHS is a method of uniquely calculating the correction coefficients from the image datasets included in the modeling method, and eFIHS-SRF is a method of uniquely calculating the correction coefficients using the relative spectral response graph. The techniques for intensity correction use two methods: the first uses the relative spectral response graph and the other uses modeling of the obtained image datasets. The comparative results show that the latter technique provides better performance. Since this method calculates the intensity correction coefficients from the observed data that include the effect of all factors, it is able to obtain a good result. For modeling techniques such as SAIHS [27], ISAIHS [25], and PRACS [31], the modeled intensity is mostly expressed as $I_{low}=\sum_{b\in B}\omega_{b}MS_{b}^{low}$ or $PAN_{low}=\sum_{b\in B}\omega_{b}MS_{b}^{low}$, where $\omega_{b}$ denotes the *b*-band correction coefficients; subsequently, some correction coefficients are acquired by its formula. While the previously used modeling techniques generally obtain correction coefficients by optimization using image datasets of satellites or observations, the proposed technique is optimized using an image dataset. The proposed method obtains the best result in the quality metrics score for all of the image datasets; in particular, the UIQI and ERGAS metrics gave consistently good results. The results show that the proposed technique reduces the spectral distortion compared to other related conventional techniques. This suggests that the model can be considered to be adequate. For the estimation of the correction coefficients, the proposed technique calculates the intensity correction coefficients for each of the observed data, thus obtaining the better numerical quality metrics than some of the other modeling techniques that calculate the intensity correction coefficients separately for each satellite. This suggests that the estimation of the correction coefficients for each image dataset is adequate because of the different observation conditions and spectral characteristics of the PAN and MS sensors.

## 5. Conclusions

This study proposed a novel model of low-spatial-resolution PAN images for pansharpening, and the intensity correction coefficients were computed using this model and the obtained image dataset. The PS image is generated using its coefficients and GIHS. The proposed model is formulated according to the characteristics of the relative spectral response graph. Due to its inclusion of subtraction, the design of this model is different from that of a conventional model. Therefore, the correction coefficients calculated for each image correspond to the observation conditions and sensor characteristics. Compared to other related methods, the proposed method demonstrated consistently good performance. These results show that the proposed model is adequate and effective for estimating the intensity of pansharpening. However, the experiments were performed only on the images obtained from IKONOS; therefore, further verification experiments on images obtained from other optical sensors are required. We would like to consider finding new application as an important future work.

## Figures and Tables

**Table 1:** Characteristics of the original and test images of the image datasets. MS: multi-spectral, PAN: panchromatic.

Image		Nihonmatsu	Yokohama	Tasmania
Original image	PAN image	1024 × 1024	1792 × 1792	12,112 × 13,136
	MS image	256 × 256	448 × 448	3028 × 3284
Test image	PAN image	256 × 256	448 × 448	3028 × 3284
	MS image	64 × 64	112 × 112	757 × 821

**Table 2:** Image size for the estimation of the intensity correction coefficients and image fusion.

Image	Estimation of Intensity Correction Coefficients	Image Fusion
PAN image $PAN^{high}$	Down-sampled Test PAN image $\left({\mathbb{R}}^{\frac{P_{row}}{R}\times \frac{P_{col}}{R}}\right)$	Test PAN image $\left({\mathbb{R}}^{P_{row}\times P_{col}}\right)$
MS images $MS_{b}^{low}$	Test MS images $\left({\mathbb{R}}^{\frac{P_{row}}{R}\times \frac{P_{col}}{R}}\right)$	Up-sampled test MS images $\left({\mathbb{R}}^{P_{row}\times P_{col}}\right)$

**Table 3:** Estimated intensity correction coefficients.

Correction Coefficient	Nihonmatsu	Yokohama	Tasmania
α (NIR)	0.3857	0.3789	0.5734
β (Blue)	0.2199	0.2549	0.2310
γ (Green)	0.1980	0.1123	0.0000
ξ (Red)	0.0486	0.1099	0.1039

**Table 4:** Correlation coefficient (CC) and university image quality index (UIQI) quality metrics results. IHS: intensity–hue–saturation, SAIHS: spectral-adjustment IHS method, ISAIHS: improved SAIHS, eFIHS-SRF: expanded fast IHS with the spectral response function, PRACS: practical replacement adaptive component substitution, BT-SFIM: brovey transform-smoothing filter based intensity modulation. The first row indicates the ideal values. For each metric, the best values are given in bold, the second-best values are underlined, and the third-best values are double underlined.

Method	CC			UIQI		
	Nihonmatsu	Yokohama	Tasmania	Nihonmatsu	Yokohama	Tasmania
Ideal value	1.0			1.0		
fast IHS	0.783	0.914	0.934	0.717	0.901	0.686
SAIHS	0.830	$\underline{\underline{0.928}}$	0.936	0.743	0.905	0.638
ISAIHS	**0.887**	**0.939**	0.949	$\underline{\underline{0.830}}$	$\underline{\underline{0.908}}$	0.708
Tradeoff IHS	0.843	$\underline{\underline{0.928}}$	0.954	0.804	0.909	$\underline{\underline{0.767}}$
eFIHS-SRF	0.818	0.914	0.949	0.733	0.825	0.821
PRACS	0.867	0.865	0.862	0.808	0.791	0.508
BT-SFIM	$\underline{\underline{0.879}}$	0.930	$\underline{\underline{0.952}}$	0.851	$\underline{\underline{0.908}}$	0.760
Proposed method	0.883	$\underline{\underline{0.928}}$	**0.966**	**0.864**	**0.910**	**0.935**

**Table 5:** *Erreur relative globale adimensionnelle de synthese* (ERGAS) and spectral angle mapper (SAM) quality metrics results.

Method	ERGAS			SAM		
	Nihonmatsu	Yokohama	Tasmania	Nihonmatsu	Yokohama	Tasmania
Ideal value	0.0			0.0		
fast IHS	3.471	$\underline{\underline{3.716}}$	11.440	1.898	2.367	2.912
SAIHS	6.653	4.772	17.775	2.198	2.339	3.932
ISAIHS	4.258	4.470	15.612	1.861	2.297	3.643
Tradeoff IHS	$\underline{\underline{2.910}}$	3.175	$\underline{\underline{8.617}}$	$\underline{\underline{1.783}}$	$\underline{\underline{2.246}}$	$\underline{\underline{2.650}}$
eFIHS-SRF	6.407	7.647	4.578	**1.638**	$\underline{\underline{2.251}}$	$\underline{\underline{2.102}}$
PRACS	2.870	4.758	8.932	1.993	3.192	5.246
BT-SFIM	4.193	4.138	9.222	2.004	2.408	2.712
Proposed method	**2.673**	**2.697**	**3.625**	1.753	**2.176**	**1.984**
